# Comparison of manual and virtual model surgery for wafer fabrication in maxillary repositioning: an in vitro study

**DOI:** 10.1186/s13005-025-00516-7

**Published:** 2025-05-11

**Authors:** Junho Jung, Jongseok Shin, Joo-Young Ohe, Byung-Joon Choi

**Affiliations:** 1 Department of Oral & Maxillofacial Surgery, Kyung Hee University College of Dentistry, Kyung Hee University Medical Center, Seoul, Republic of Korea; 2 Department of Dentistry, Graduate School, Kyung Hee University, Seoul, Republic of Korea

**Keywords:** Manual model surgery, Virtual model surgery, Wafer fabrication, Maxillary repositioning, Orthognathic surgery

## Abstract

**Background:**

The aim of this study was to compare the accuracy of 3D-printed intermediate wafers (3DW) with conventionally made intermediate wafers (CW) fabricated through manual model surgery (MMS). This study was designed as an in vitro experiment focused on the Le Fort I osteotomy and maxillary repositioning process. It aims to achieve maxillary repositioning outcomes mediated by intermediate wafers while eliminating intraoperative errors.

**Materials and methods:**

Both MMS and virtual model surgery (VMS) were performed for each patient to fabricate CW and 3DW. Subsequently, the maxillomandibular dental casts were remounted on the articulator using the fabricated CW and 3DW, followed by digital scanning and superimposition. The midpoint of the right maxillary central incisor edge, the uppermost points of the right and left maxillary canines, and the mesiobuccal cusps of the right and left maxillary molars were used as measurement points. The points in VMS were set as references for comparison. Paired *t*-tests were conducted to compare the outcomes between CW and 3DW. Independent *t*-tests were used to analyze differences between groups with and without rotational movements. Additionally, Spearman’s correlation analysis was performed to examine the relationship between the rotational movement of the maxilla and the corresponding coordinate differences.

**Results:**

Significant differences were observed in the transverse (*p* = 0.005), anteroposterior (*p* = 0.016), and vertical (*p* = 0.003) coordinates between the maxillary positions derived from CW and VMS. In MMS, the presence of roll movement significantly influenced transverse position (*p* = 0.002), pitch affected vertical position (*p* < 0.001), and yaw impacted transverse (*p* = 0.005) and vertical (*p* = 0.019) positions.

**Conclusion:**

3DW demonstrated greater accuracy than MMS with CW. Especially in cases involving rotational maxillary movements such as roll, yaw, and pitch, it resulted in fewer errors compared to MMS with CW. Consequently, 3DW offers more precise recording of maxillary repositioning plan and contributes to the successful transfer of this plan into the surgical outcome in orthognathic surgery.

**Supplementary Information:**

The online version contains supplementary material available at 10.1186/s13005-025-00516-7.

## Introduction

Le Fort I osteotomy is the most frequently performed procedure for repositioning the maxilla in orthognathic surgery. Over the years, numerous surgeons have refined their intraoperative techniques and preoperative planning to accurately reposition the maxilla as needed [1, 2]. Intraoperatively, precise maxillary repositioning is facilitated by both internal and external measuring systems [2]. However, the success of this procedure heavily relies on a preoperatively fabricated surgical wafer that records the planned position of the maxilla. Therefore, conducting model surgery or virtual surgery to reposition the maxilla and fabricate an intermediate wafer in the laboratory is a critical step in orthognathic surgery. This process not only guides the three-dimensional (3D) movement of the maxilla but also ultimately determines the position of the mandible.

Manual model surgery (MMS), the traditional technique for manipulating the 3D movements of the maxillary dental cast, is commonly used in preoperative laboratory procedures for orthognathic surgery [3]. MMS primarily depends on the facebow transfer technique and the mounting of dental casts onto an articulator. To accurately replicate the original position of the maxilla and ensure the reliable movement of the dental cast, various specialized instruments and methods have been developed [4–7]. However, reports indicate that these methods still have potential errors [1, 5, 6, 8]. In particular, inaccurate setting of the Frankfort Horizontal plane can introduce technical errors, as maxillary movement in three-dimensional axes is based on this plane [9]. Reference points and lines drawn manually are also subject to human error, making MMS inevitably dependent on the operator’s expertise. The plane used in MMS inevitably differs from that in VMS, which is established based on skull data. Reference points and lines drew by analog way also possess human errors. Therefore, it makes MMS inevitably dependent on the expertise of operator and require a high level of proficiency. Furthermore, MMS is time-consuming involves several complex laboratory steps. Consequently, the intermediate wafer fabricated through MMS inherently accumulates errors from each preceding step [5, 10]. Before the emergence of 3D technology, MMS with hand-made intermediate wafers was the most prevalent method for preparing for orthognathic surgery [6, 10]. 

Currently, computer-aided technology is integral to the preparation for orthognathic surgery [8, 11–13]. A computed tomography (CT) scan of the skull is utilized to create 3D cephalometric measurements and facilitate virtual surgical simulations [10, 14–16]. Instead of manually manipulating dental casts and employing traditional methods to fabricate intermediate wafers, 3D Virtual Model Surgery (VMS) and stereolithographic intermediate wafers have emerged as alternative approaches for preparing orthognathic surgeries [6, 8, 10, 17, 18]. The VMS program enables precise repositioning of the maxilla, reduces laboratory effort, and shortens fabrication time [10]. Additionally, it serves as a decision-support tool by visualizing postoperative outcomes [10]. However, there are reports indicating that the 3D fabrication process may lead to technical errors [6, 10]. 

Several studies have explored the viability of VMS and the accuracy of digitally fabricated wafers concerning surgical outcomes [3, 8, 11, 17, 18]. These studies have evaluated the accuracy of the repositioned maxilla by comparing pre- and post-surgical cephalometric landmarks [1, 3, 8, 15, 19, 20]. A significant challenge in comparing preoperative plans with postoperative results, as noted in previous studies, is that intraoperative factors can greatly influence surgical outcomes and introduce potential errors. This is particularly pertinent in Le Fort I osteotomy and repositioning of the maxilla using an intermediate wafer, as these depend on the positioning of the mandibular condyle, which is controlled by the surgeon during the procedure. Additionally, the maxilla might be distorted by the plating procedure after the application of surgical wafers, preventing the postoperative maxillary position from fully reflecting the influence of the wafers. Furthermore, for an accurate comparison, conventionally fabricated and digitally fabricated wafers should be simultaneously prepared and compared within a single patient.

To eliminate the influence of the intraoperative factors and solely evaluate the maxillary position determined by surgical wafers, the two types of wafers were prepared for each patient in this study: 3D-printed intermediate wafers (3DW) created using VMS, and conventionally made intermediate wafers (CW) fabricated through MMS. Afterwards, maxillary repositioning procedures were then mimicked through in vitro model experiments by re-mounting the maxillomandibular dental casts using the wafers. The final maxillary positions derived from both types of wafers were digitally scanned and compared three-dimensionally to the positions preoperatively planned by VMS.

## Materials and methods

### Study design/sample

This study involved data from 15 individuals (9 men and 6 women; mean age, 22.8 ± 2.3 years) who had sought orthognathic surgery at the Department of Oral and Maxillofacial Surgery at Kyung Hee University between 2020 and 2022. Each patient had undergone a Le Fort I osteotomy for the maxilla and a bilateral sagittal split ramus osteotomy for the mandible. Since simpler movements involving one-way movement of the maxilla has a lower potential for error during model surgery, we selected data from patients who exhibited at least two types of movement among anteroposterior, transverse, vertical, roll, pitch, and yaw for maxillary repositioning. Roll rotation is defined as the rotation of the maxilla around its longitudinal axis (anteroposterior axis), occurring when there is a discrepancy between the left and right tooth vertical positions. Pitch rotation is defined as the rotation of the maxilla around its side-to-side axis (transverse axis), occurring when there is a discrepancy between the anterior and posterior tooth vertical positions. Yaw rotation is defined as the rotation of the maxilla around its vertical axis, occurring when there is a discrepancy between the left and right tooth transverse and anteroposterior positions.

Additionally, surgical plans involving downward movement of the maxilla were not included, as these require a mandibular opening in VMS and MMS to eliminate dental interference and ensure the thickness of the wafers.

**Fig. 1:**
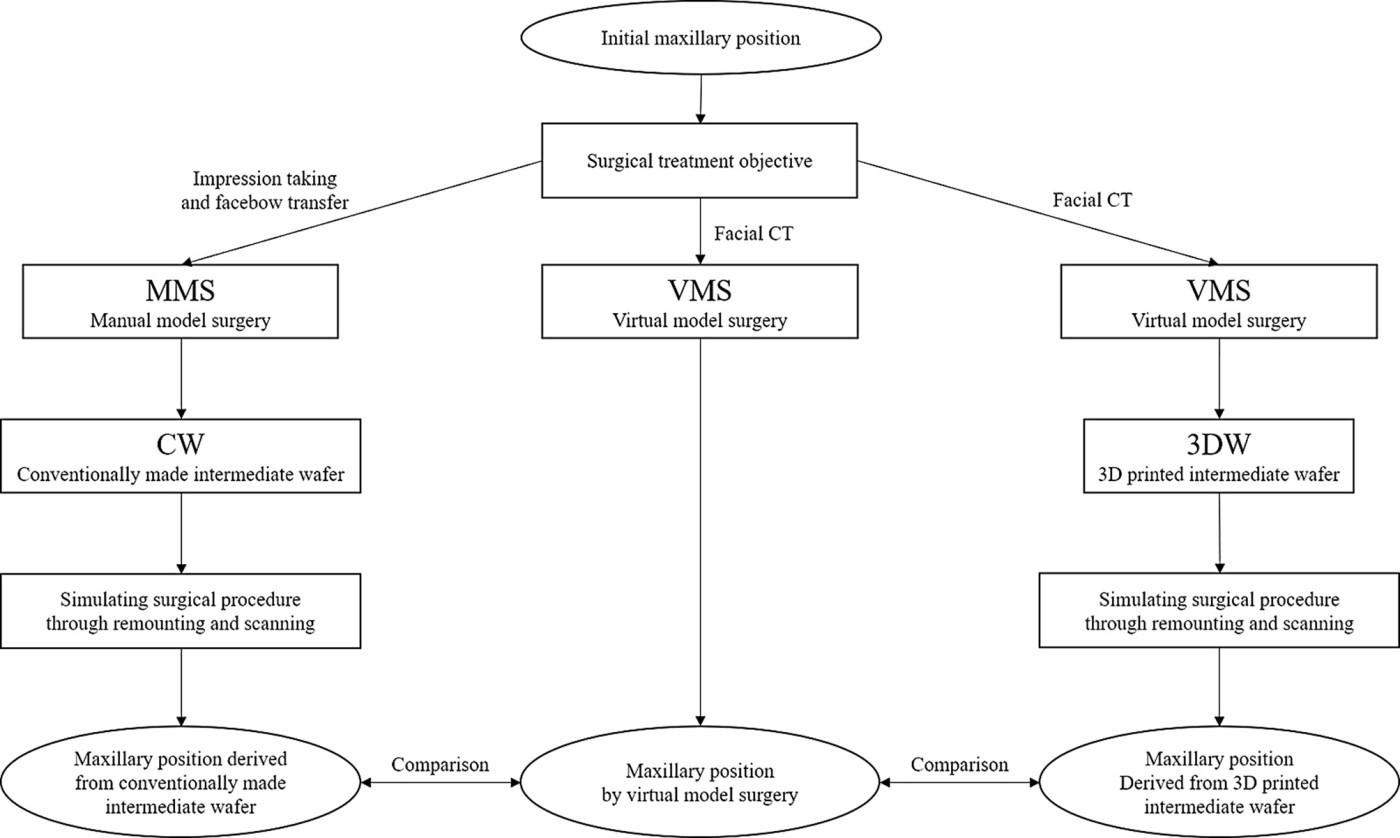
Flowchart of the experimental procedure involving manual model surgery, wafer fabrication, and remounting of the maxillary dental cast

For each patient, CT scans and dental impressions had been taken one month prior to surgery. When taking a CT scan, a bite registration recorded for the centric relation position of the mandible was used, allowing for approximately a 1 mm opening between the maxillary and mandibular molars. Surgical treatment objectives (STOs) had been established using 3D cephalometric measurements (Mimics; Materialise, Leuven, Belgium) at the time of surgery. Their treatment objectives were reflected into this study, and their original wafers were not used. To create a 3D model of the skull, the threshold value was set at 226 Hounsfield Units (HU) or higher. Model surgery was performed using both MMS and VMS (Mimics). Subsequently, two types of wafers were fabricated for each patient: CW from MMS and 3DW from VMS. The amount of maxillary and tooth movement used for MMS was also calculated through VMS complying with the STO. The amount of the tooth movements was presented in supplementary Table 1. The experimental procedures for model surgery, wafer fabrication, and the remounting process are illustrated in Fig. 1.

This study was approved by the Ethics Committee of Kyung Hee University Dental Hospital (KH-DT22022).

### Simulation design

#### MMS and CW

**Fig. 2:**
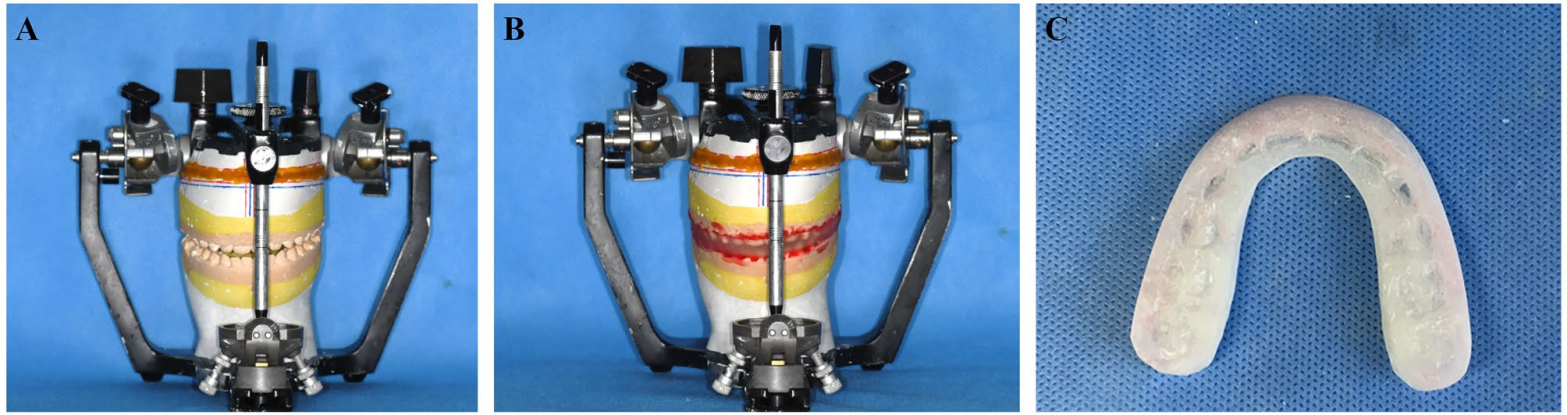
(**A**, **B**) After manual model surgery, a set of maxillary and mandibular casts was mounted onto the articulator, followed by the fabrication of a conventional intermediate wafer. (**C**) Conventionally fabricated intermediate wafer

A set of maxillomandibular dental casts from each patient was mounted on a semi-adjustable articulator (Hanau, Buffalo, NY, USA) using a facebow (Hanau, Buffalo, NY, USA) transferring the FH plane and a centric relation bite registration, which is the same bite used for CT scans. The maxillary dental cast was then removed from the articulator and placed on a model surgery platform (SP orthodontics, Seoul, Republic of Korea) to record the coordinates of the measurement points and to conduct the reposition of the casts. The MMS procedure was carried out as follows:

1. Original Coordinate Measurement: The initial maxillary position with reference points was measured using a digital caliper (Mitutoyo, Kanagawa, Japan). Measurements included the transverse position (x-axis), anteroposterior position (y-axis), and vertical position (z-axis) of the midpoint of the right maxillary central incisor edge, along with the uppermost points of the right and left maxillary canines and the mesiobuccal cusps of the right and left maxillary molars.
2. Manual Repositioning of the Maxillary Cast: The maxillary cast was manually repositioned according to the tooth position measured in VMS. Acknowledging the limitations of 3D imaging software, we utilized only the differences in coordinates across the three axes between the original and repositioned landmarks (Fig. 2). The relocation process of the maxillary cast was continued until the discrepancy between the target values and the measured values was less than 0.1 mm. Once the maxillary dental cast was repositioned, the reference points were measured again for confirmation.

3. Fabrication of the Conventional Wafer: The repositioned maxillary dental cast was attached to the articulator. CW was then fabricated using orthodontic acrylic resin (Orthojet; Land Dental Manufacturing, Wheeling, IL, USA). The wafers for all 15 patients were fabricated and trimmed in preparation for the remounting procedure.

#### VMS and 3DW

**Fig. 3:**
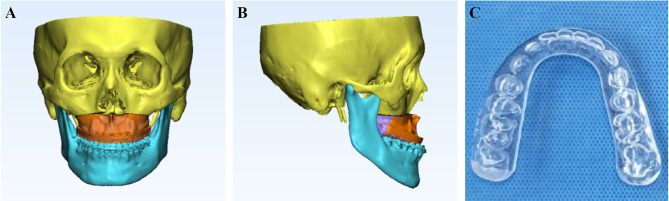
(**A**, **B**) Fontal and lateral views of the virtual model surgery. The initial maxillary position is highlighted in purple, while the repositioned maxillary position is shown in orange. (**C**) 3D-printed intermediate wafer

The procedure of VMS and the production of the intermediate wafer using a 3D printer were as follows:

1. 3D Scanning and Superimposition: Preoperative maxillary and mandibular casts were scanned using a 3D laser scanner (MDS500; Maestro3D, Pontedera, Italia). The scanned 3D dental models were then superimposed onto CT scans using 3D imaging software for VMS.
2. Virtual Repositioning: Using the Frankfort Horizontal Plane as a reference, the maxilla was virtually repositioned based on the STOs using Mimics software (Fig. 3).

3. Wafer Design and Printing: After confirming the coordinates of the final maxillary position through VMS, wafers were designed and printed using a 3D printer (Projet 6000; 3D Systems, Rock Hill, SC, USA) with photoactivated resin (Accura SI 40 Nd-type stereolithography resin; 3D Systems; Fig. 3). The printer achieves an accuracy ranging from 0.001 to 0.002 inches per inch of part dimension, with a layer thickness of 0.1 mm [21]. Wafers for all 15 patients were fabricated and trimmed in preparation for the remounting procedure.

### Remounting and scanning for superimposition

**Fig. 4:**
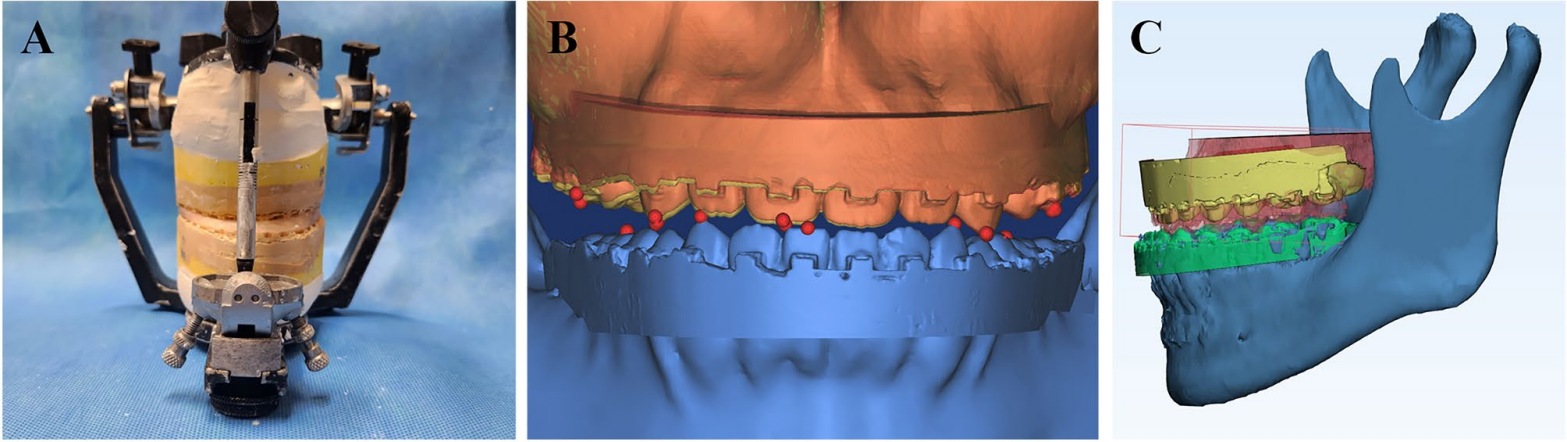
(**A**) Remounting the set of maxillomandibular casts using CW and 3DW. (**B**) Reference points for the measurement of maxillary movement. (**C**) Superimposition of the maxillomandibular dental casts based on the mandibular position

**Fig. 5:**
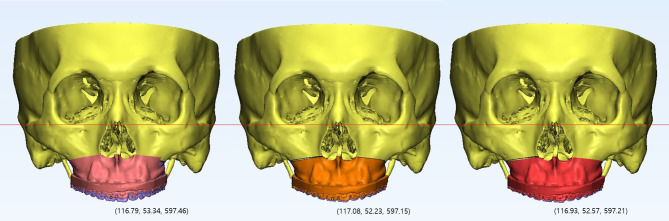
Measurement of reference points in the relocated maxillary position determined by the conventionally fabricated wafer (left), virtual model surgery (middle), and 3D-printed wafer (right). The coordinates of the midpoint of the upper right incisor edges are displayed for each final maxillary position

To mimic the Le Fort I osteotomy and maxillary repositioning procedures in the laboratory, the maxillary casts were remounted using both CW and 3DW, and the position of the maxilla was measured and compared. The remounting procedure consisted of the following steps:

1. Preoperatively taken maxillary and mandibular casts were used for remounting.
2. After positioning the mandibular dental casts on the articulator, the maxillary dental cast was secured in place using CW (Fig. 4) and subsequently remounted.

3. Following the removal of the CW, the mandibular dental cast, the maxillary dental cast, and their interocclusal relation were scanned with a 3D laser scanner (Orapix).
4. Using 3D imaging software (Mimics; Materialise, Leuven, Belgium) and utilizing data from VMS, the scanned maxillomandibular dental casts, along with their interocclusal relation, were superimposed onto the mandibular dentition (Fig. 5). For consistent comparison of the final maxillary position, the mandibular dentition served as the reference.

The accuracy of the repositioned maxillary coordinates was assessed by comparing the predetermined measurement points in VMS. These points included the right maxillary central incisor, both right and left maxillary canines, and the first molars on both sides. The coordinates of these points were marked and recorded during the remounting and scanning procedures for comparison, as described above. A smaller discrepancy in the transverse, anteroposterior, and vertical positions indicated a more precise result.

To ensure consistency, the measurement of coordinates in the 3D simulation software was repeated twice by two examiners with a two-week interval. The ICC values were 0.793 for inter-rater reliability and 0.910 for intra-rater reliability.

### Statistical analysis

To evaluate the positional differences of the maxilla between the reference position in VMS and the positions in CW and/or 3DW, paired *t*-tests were conducted. The data was divided into groups based on the presence or absence of rotation, and independent *t*-tests were used to compare the coordinate differences between the groups. For non-normally distributed data, Mann–Whitney U test with were applied. Additionally, Spearman’s correlation was performed to examine the relationship between the rotational movement of the maxilla and the corresponding coordinate differences across various types of maxillary movements. Statistical significance was set at *p* < 0.05, and analyses were performed using SPSS 27.0 (IBM Corp., Armonk, NY, USA).

## Results

### Comparison of maxillary positions derived from conventionally fabricated wafer, 3D printed wafer, and virtual model surgery

The mean coordinates for the maxillary measurement points from CW in the transverse, anteroposterior, and vertical positions were 116.28 ± 22.89, 58.26 ± 13.27, and 578.30 ± 27.31, respectively. VMS recorded mean coordinates of 116.03 ± 22.83, 57.90 ± 13.49, and 578.02 ± 27.27 in the same respective positions. Statistical comparisons between the maxillary positions derived from CW and VMS demonstrated significant differences in the transverse (*p* = 0.005), anteroposterior (*p* = 0.016), and vertical (*p* = 0.003) coordinates (Table 1).

**Table 1:** Comparison between the maxillary positions from CW and 3DW based on VMS

		Tooth position	Mean	SD	Sig.
Transverse (x)	VMS ^a^	#11	112.95	7.97	
		#13	98.44	8.31	
		#23	135.68	7.71	
		#16	88.58	7.72	
		#26	144.50	8.53	
	CW ^b^ (Δx)	#11	0.61	0.53	0.005*
		#13	0.62	0.48	
		#23	0.61	0.62	
		#16	0.60	0.53	
		#26	0.59	0.41	
	3DW ^a^ (Δx)	#11	0.14	0.11	0.499
		#13	0.20	0.10	
		#23	0.10	0.09	
		#16	0.11	0.11	
		#26	0.18	0.15	
	VMS ^a^	#11	44.51	9.34	
		#13	52.54	9.02	
		#23	52.87	8.49	
		#16	68.73	9.38	
		#26	70.83	8.86	
Anteroposterior (y)	CW ^b^ (Δy)	#11	0.96	0.92	0.016*
		#13	1.06	1.05	
		#23	1.03	0.71	
		#16	1.00	1.00	
		#26	0.96	0.70	
	3DW ^a^ (Δy)	#11	0.11	0.09	0.621
		#13	0.19	0.12	
		#23	0.18	0.14	
		#16	0.16	0.10	
		#26	0.20	0.14	
	VMS ^a^	#11	580.67	27.61	
Vertical (z)		#13	579.28	27.56	
		#23	579.46	27.11	
		#16	575.18	28.99	
		#26	575.53	28.42	
	CW ^b^ (Δz)	#11	0.54	0.31	0.003*
		#13	0.63	0.36	
		#23	0.88	0.38	
		#16	0.70	0.36	
		#26	0.81	0.38	
	3DW ^a^ (Δz)	#11	0.12	0.07	0.533
		#13	0.13	0.09	
		#23	0.16	0.12	
		#16	0.16	0.18	
		#26	0.14	0.09	

The same superscript letter indicates statistical insignificance, and different letters indicates statistical significance. *p* < 0.05 was considered statistically significant. Asterisks indicate statistical significance compared to VMS. CW, conventionally fabricated wafer; 3DW, 3D-printed wafer; VMS, virtual model surgery; SD, standard deviation

**Fig. 6:**
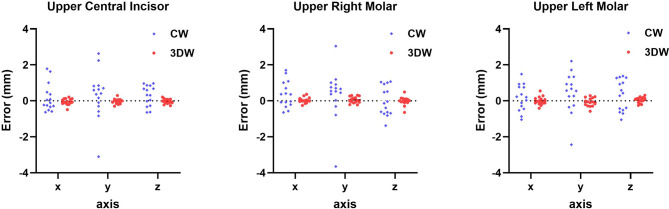
Scatter plot showing the discrepancies in the three axes (transverse, anteroposterior, and vertical) of measurement points between CW and 3DW

The mean coordinates for the maxillary measurement points from 3DW in the transverse, anteroposterior, and vertical positions were 116.04 ± 22.82, 57.88 ± 13.48, and 578.01 ± 27.28, respectively. It was statistically insignificant compared to those from VMS (*p* = 0.499, 0.621, and 0.533, respectively). However, when comparing the maxillary positions derived from CW and 3DW, significant differences were observed in the transverse (*p* = 0.010), anteroposterior (*p* = 0.009), and vertical (*p* = 0.003) coordinates (Fig. 6).

### Influence of rotational maxillary movements on positional errors in maxillary reposition

**Fig. 7:**
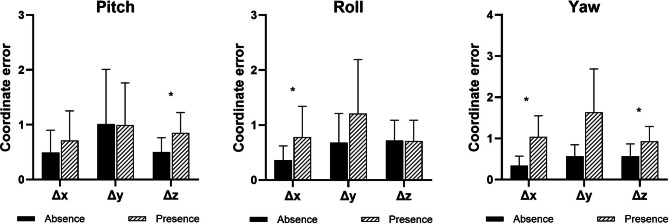
Influence of rotational maxillary movements. Coordinate differences (mm) between the repositioned maxillary position derived from the conventionally fabricated wafer and VMS were measured. Error bars indicate the standard deviation. Asterisks (*) indicate statistically significant differences compared to the group without rotational movement (*p* < 0.05)

To investigate the influence of rotational movements of the maxilla on STOs, coordinate differences in the transverse, anteroposterior, and vertical positions were analyzed based on the planned roll, pitch, and yaw movements for the subjects. Notably, the presence of rotational maxillary movements significantly impacted the coordinate differences between CW and VMS (Table 2). Specifically, roll movement significantly influenced the transverse position (*p* = 0.002) derived from CW compared to that derived from VMS (Fig. 7). Additionally, pitch movement significantly affected the vertical position (*p* < 0.001). Yaw movement also significantly impacted the transverse (*p* = 0.005) and vertical (*p* = 0.019) maxillary positions derived from CW. However, Spearman’s correlation analysis showed that the amount of roll movement planned on STO was not significantly correlated with transverse positional errors (r_s_ = 0.212). Similarly, the amounts of pitch and yaw movements were not significantly correlated with the vertical and transverse positional errors of the maxilla derived from CW (r_s_ = 0.385 and 0.537, respectively). Additionally, planar movements like advancement, translation, and impaction of the maxilla on STO did not significantly impact any of the measured coordinates.

**Table 2:** Influence of rotational movements on coordinate differences of the maxilla between CW and VMS

Types of rotational movements		Transverse (Δx)	Anteroposterior (Δy)	Vertical (Δz)
pitch	Absence	0.49 ± 0.41	1.01 ± 1.00	0.50 ± 0.26
	Presence	0.71 ± 0.54	0.99 ± 0.77	0.85 ± 0.37*
roll	Absence	0.36 ± 0.26	0.68 ± 0.53	0.72 ± 0.37
	Presence	0.78 ± 0.56*	1.21 ± 0.98	0.71 ± 0.38
yaw	Absence	0.34 ± 0.23	0.57 ± 0.28	0.57 ± 0.30
	Presence	1.04 ± 0.51*	1.64 ± 1.05	0.93 ± 0.36*

*p* < 0.05 was considered statistically significant. Asterisks (*) indicate statistically significant differences compared to the group without rotational movements. CW, conventionally fabricated wafer; VMS, virtual model surgery

## Discussion

Previously, surgical wafers fabricated by conventional methods and 3D printing were compared using radiographic data of pre- and postoperative maxillary positions [1, 3, 8, 15, 19, 20]. However, postoperative outcomes were significantly influenced by intraoperative surgical factors. Since the positioning of the maxilla with intermediate wafers is highly dependent on the manipulation of the maxillomandibular complex and condylar position, it is essential to isolate surgeon-related factors to accurately assess the effectiveness of intermediate wafers. Additionally, intraoperative adjustments to the wafers relative to the dentition and forces applied during maxillomandibular fixation can interfere with the accurate reflection of preoperative plans recorded to the wafers. Moreover, the process of adapting screws and plates may further distort the final position of the maxilla.

To address this, additional steps—including remounting, scanning, and subsequent superimposition—were introduced to mimic maxillary repositioning in orthognathic surgery, thereby eliminating intraoperative influences. Additionally, for each patient, we conducted both MMS and VMS, allowing us to assess two types of surgical wafers simultaneously, which is another advantage of this simulation [3, 8, 19, 20]. Through these steps, we were able to simulate the actual adaptation of wafers and analyze their influence on surgical outcomes, not measuring the thickness of the wafers.

Significant differences were observed across all three 3D coordinate axes of the maxillary position between CW and 3DW. While no significant differences between 3DW and VMS were observed, positional errors in all three directions were associated with CW compared with VMS. As hypothesized, CW fabrication following MMS was more prone to errors. During the preoperative planning for maxillary movement in orthognathic surgery, both planar movements and rotational movements (e.g., roll, pitch, and yaw) were considered. These rotational movements of the maxilla are challenging to reproduce during MMS, potentially leading to greater positional errors. Our results indicated significant differences in the transverse coordinates for roll and the vertical coordinates for pitch movement. Additionally, the presence of yaw in surgical planning increased the likelihood of positional errors in all coordinates. Yaw movement requires a high level of expertise for accurate maxillary cast repositioning, as it involves simultaneous adjustments in the transverse and anteroposterior coordinates while maintaining vertical coordinates during the manual manipulation of the maxillary cast. Furthermore, yaw adjustment in MMS is particularly challenging due to the absence of a consistent reference point, requiring reliance on reference lines that are difficult to digitize. Currently, devices used in MMS are primarily designed to measure the height of reference points. This limitation likely contributes to the increased potential for errors associated with the presence of yaw movements. However, the correlation between the extent of the movement and the errors was not identified. To consolidate the findings and achieve more credible results, a larger sample size is needed.

Other studies have also supported the accuracy of 3D methods in orthognathic surgery. One study demonstrated that 3D virtual computer-assisted planning offers higher accuracy compared to traditional methods like facebow transfer [9]. Another study investigated the accuracy of digitally fabricated intermediate wafers and found that the surgical outcomes were statistically insignificant from the planned maxillary movement in three dimensions [22]. In a randomized controlled study comparing digital and conventional resin wafers, digital wafers showed superior outcomes in transferring the surgical plan to the operation environment Additionally, a study comparing the validity of 3D-printed wafers and CW demonstrated that 3D-printed wafers is acceptably accurate in three spatial dimensions, in both laboratory and clinical settings [23]. Reported errors from other studies during the laboratory steps of VMS and 3DW ranged from 0.03 to 1.4 mm [6] and from 0 to 0.35 mm, respectively [10]. 

MMS has traditionally been the standard method for fabricating intermediate wafers. Despite efforts to enhance the accuracy of dental cast movement in MMS [4–7, 24, 25], manual manipulation during model surgery still results in three-dimensional inaccuracies. Technical errors may also arise during impression-taking, facebow transfer, and the mounting process [1, 8, 9]. Improper dental impressions can reduce the fit of wafers to dentition, which in turn deteriorates the accuracy of maxillary repositioning. Additionally, errors during the facebow transfer can lead to an incorrect Frankfort Horizontal plane orientation. Consequently, the three-dimensional coordinates of the maxillary cast may differ from those oriented in VMS. Moreover, the three-dimensional movement of the maxillary cast during MMS is highly dependent on the operator’s expertise. Reference lines for the maxillary movement, illustrated on the cast, inherently possess human errors and are challenging to accurately align with the three-dimensional axes set by the Frankfort Horizontal plane. Since 3D fabrication of wafers is directly accomplished from VMS, several steps prone to technical errors are eliminated, except the superimposition process, which involves merging scan data from dental casts and CT scans, can be a major origin of the errors. A study demonstrated that five out of six directions of maxillary movement in the articulator exhibited errors exceeding 1 mm, with inaccuracies in midline repositioning leading to corresponding discrepancies in the mediolateral repositioning of the posterior section of the maxilla [5]. Manually repositioning the maxillary dental cast to the precise 3D location, especially in cases involving yaw, presents significant challenges for the operator. Furthermore, these complex laboratory procedures in MMS are not only time-consuming but also require considerable effort.

Several studies have reported on the accuracy of 3D virtual datasets generated through VMS and laser scanning techniques. However, potential errors during the acquisition of virtual datasets in VMS have also been noted [26–28]. Limitations of CT imaging, including inadequate noise levels, resolution, contrast, and image quality, hinder the establishment of a precise digital mounting method for VMS [13]. Furthermore, the slicing thickness of CT data may limit the accuracy of occlusal surface reconstruction, affecting the precision of reference points in the digital mounting process on programmed articulators and potentially introducing errors. Artifacts at the occlusal level of dentition in CT imaging further compromise the accuracy of occlusal and intercuspidation data.

Fully automated and point-based semi-automated superimposition technologies have been developed to merge scan data from dental casts and CT scans to enhance the resolution of dentition [29–31]. However, these technologies do not guarantee accuracy, particularly in cases involving orthodontic braces, which may be inadequately represented due to metal artifacts in CT scans. Therefore, manual adjustment of scanned dentition to align with CT-derived dentition is necessary, inherently introducing human errors even within digital processes. Additionally, errors may occur during the wafer fabrication process with 3D printers. Stereolithographic technology, which is still under development, can introduce errors due to dimensional changes in the resin from sequential curing and the removal of residual resin using alcohol [10]. 

The results of this study suggest that 3DW with VMS offers greater accuracy than CW with MMS, as confirmed by in vitro simulations that excluded intraoperative factors. 3DW facilitates more precise alignment of the maxilla as planned in VMS, particularly when rotational movements of the maxilla, such as yaw, roll, and pitch, are required in maxillary repositioning. The study demonstrates that manually reproducing the preoperatively planned maxillary position is considerably challenging. Therefore, it is recommended to directly fabricate surgical wafers using a 3D printer, as planned and designed through 3D imaging solutions, to achieve accuracy, despite potential technical errors arising from digital scanning, superimposition, and printing. It also reduces laboratory work and time for the operator, thereby decreasing overall effort. In the foreseeable future, advancements in 3D imaging software and 3D printing technology might enable VMS and 3D printing to entirely replace MMS. However, it must be noted that there are inherent errors originating from the 3D software. The errors from the scanning process of dental casts and the merging of scan data from dental casts and CT scans are inevitable. These errors may favor the results from 3DW, as the maxillary position set in VMS was used as a standard. Moreover, the costs associated with the laboratory setup for 3D software, printers, and scanning devices are significantly higher than those for CW. Even when virtual planning and 3D printing is outsourced to external laboratories, the expenses remain relatively higher. Additionally, the availability of 3D solutions can vary greatly depending on the region.

This study isolated surgical factors that influence maxillary repositioning and mimicked the relocation process in vitro, similar to Le Fort I osteotomy. However, a larger sample size is necessary for a thorough analysis of the factors contributing to inaccuracies in wafer fabrication. Furthermore, it is important to acknowledge that more skilled technicians may enhance the accuracy of MMS. Depending on the expertise of the MMS operator, the gap between CW and 3DW may not be significant. Particularly in cases involving complex movements of the maxilla, such as rotational movements, the likelihood of errors may greatly depend on the operator’s experience. Therefore, conducting a study to compare outcomes from various MMS operators is crucial to quantify this influence. Additionally, the post-processing of 3D-printed wafers can result in varying levels of accuracy. It is also important to note that different types or brands of 3D printers and printing materials are reported to have varying accuracies [21, 32]. Recently, the accuracy of intraoral scanners has improved significantly. While we utilized a laboratory model scanning device, direct dentition scanning from patients could simplify the preoperative work-up for orthognathic surgery by eliminating the need for dental impressions. However, current complete-arch scanning with intraoral scanners is reported to be insufficiently accurate for fixed appliances [33–35]. The impact of this limitation on the fabrication of wafers and maxillary repositioning in orthognathic surgery should be investigated in future studies. Furthermore, newer acquisition technologies may enhance accuracy and improve these outcomes [35]. 

## Electronic supplementary material

Below is the link to the electronic supplementary material.

Supplementary Material 1

## Data Availability

No datasets were generated or analysed during the current study.
