# Supplementary material for: Comparison of manual and virtual model surgery for wafer fabrication in maxillary repositioning: an in vitro study
Source: Head Face Med. 2025 May 11;21:38. doi: 10.1186/s13005-025-00516-7 (PMC12066060; doi:10.1186/s13005-025-00516-7)
Supplement: Supplementary file 1 — Supplementary Material 1 [file 13005_2025_516_MOESM1_ESM.docx]

**Supplementary Table 1**. Amount of maxillary movements according to tooth position as calculated through VMS

| Patient ID | Right Maxillary 1^st^ Molar | | | | Right Central Incisor | | | | Left Maxillary 1^st^ Molar | | |
| --- | --- | --- | --- | --- | --- | --- | --- | --- | --- | --- | --- |
|  | Transverse (x) | A-P (y) | Vertical (z) | Transverse (x) | | A-P (y) | Vertical (z) | Transverse (x) | | A-P (y) | Vertical (z) |
| 1 | 1.16 | -0.35 | 3.59 | 0.01 | | -0.96 | 0.52 | 0.84 | | -2.38 | 3.41 |
| 2 | 0.01 | -2.22 | 3.99 | -0.01 | | -2.04 | 3.16 | -0.01 | | -1.78 | 2.01 |
| 3 | 2.55 | -3.51 | 3.16 | 1.04 | | -2.73 | 0.09 | 2.45 | | -3.40 | 2.84 |
| 4 | 0.00 | -2.02 | 2.96 | 0.00 | | -1.91 | -0.02 | 0.00 | | -1.90 | 3.04 |
| 5 | 0.71 | 1.02 | 1.23 | 0.03 | | 0.28 | 0.97 | 1.29 | | -1.71 | 4.07 |
| 6 | 1.00 | -1.19 | 2.40 | 1.00 | | -0.91 | -0.01 | 1.00 | | -0.98 | 2.60 |
| 7 | -0.53 | -0.74 | 3.30 | -0.02 | | -0.03 | 0.51 | -0.47 | | 0.42 | 1.50 |
| 8 | 0.48 | 0.43 | 3.00 | -0.02 | | 0.08 | 2.59 | 0.52 | | -0.44 | 2.00 |
| 9 | 1.50 | -0.35 | 0.97 | 1.50 | | 0.01 | 0.00 | 1.50 | | -0.28 | 1.03 |
| 10 | -0.02 | 0.32 | 1.01 | 0.00 | | 0.05 | 2.26 | 0.02 | | -0.32 | 3.99 |
| 11 | -1.04 | -5.93 | 0.01 | 2.84 | | -3.84 | 1.62 | 0.04 | | -0.23 | 3.99 |
| 12 | 0.99 | -4.32 | 0.01 | 1.00 | | -4.47 | 0.63 | 1.01 | | -4.68 | 1.49 |
| 13 | -0.03 | -1.23 | 1.99 | -0.03 | | -1.04 | 1.16 | 0.03 | | -0.77 | 0.01 |
| 14 | 0.00 | -2.00 | 1.00 | 0.00 | | -2.00 | 1.00 | 0.00 | | -2.00 | 1.00 |
| 15 | 0.00 | -0.15 | 3.52 | 0.00 | | 0.07 | 2.02 | 0.00 | | -0.13 | 3.48 |
